# Comparison of hemodynamic effects and resuscitation outcomes between automatic simultaneous sterno-thoracic cardiopulmonary resuscitation device and LUCAS in a swine model of cardiac arrest

**DOI:** 10.1371/journal.pone.0221965

**Published:** 2019-08-30

**Authors:** Kyoung-Chul Cha, Hyung Il Kim, Yong Won Kim, Gyo Jin Ahn, Yoon Seob Kim, Sun Ju Kim, Jun Hyuk Lee, Sung Oh Hwang

**Affiliations:** 1 Department of Emergency Medicine, Yonsei University Wonju College of Medicine, Wonju, Republic of Korea; 2 Department of Emergency Medicine, Dankook University, College of Medicine, Cheonan, Republic of Korea; 3 Department of Emergency Medicine, University of Ulsan College of Medicine, Ulsan University Hospital, Ulsan, Republic of Korea; 4 Department of Biostatistics, Yonsei University Wonju College of Medicine, Wonju, Republic of Korea; European University Cyprus, CYPRUS

## Abstract

**Introduction:**

Mechanical cardiopulmonary resuscitation (CPR) devices are widely used to rescue patients from cardiac arrest. This study aimed to compare hemodynamic effects and resuscitation outcomes between a motor-driven, automatic simultaneous sterno-thoracic cardiopulmonary resuscitation device and the Lund University cardiac arrest system (LUCAS).

**Material and methods:**

After 2 minutes of electrically induced ventricular fibrillation (VF), Yorkshire pigs (weight 35–60 kg) received CPR with an automatic simultaneous sterno-thoracic CPR device (X-CPR group, n = 13) or the Lund University cardiac arrest system (LUCAS group, n = 12). Basic life support for 6 minutes and advanced cardiovascular life support for 12 minutes, including defibrillation and epinephrine administration, were provided. Hemodynamic parameters and resuscitation outcomes, including return of spontaneous circulation (ROSC), 24-hour survival, and cerebral performance category (CPC) at 24 hours, were evaluated.

**Results:**

Hemodynamic parameters, including aortic pressures, coronary perfusion pressure, carotid blood flow, and end-tidal carbon dioxide pressure were not significantly different between the two groups. Resuscitation outcomes were also not significantly different between the groups (X-CPR vs. LUCAS; rate of ROSC: 31% vs 25%, p = 1.000; 24-hour survival rate: 31% vs 17%, p = 0.645; neurological outcome with CPC ≤2: 31% vs 17%, p = 0.645). Also no significant difference in incidence complications associated with resuscitation was found between the groups.

**Conclusions:**

CPR with a motor-driven X-CPR and CPR with the LUCAS produced similar hemodynamic effects and resuscitation outcomes in a swine model of cardiac arrest.

## Introduction

Sudden cardiac arrest is a major healthcare issue worldwide, but the survival rate remains low [1]. The quality of cardiopulmonary resuscitation (CPR) is well known to be a major determinant of survival and favorable neurological outcome in patients with cardiac arrest [2–4]. During the treatment of patients with cardiac arrest, a substantial period of resuscitation is often required to achieve return of spontaneous circulation (ROSC) [5]. Recent CPR guidelines recommend changing rescuers every 2 minutes and use of a feedback device for monitoring CPR quality [2–4]. However, high-quality CPR is difficult to maintain especially in prolonged cardiac arrest, even by well-trained rescuers [6–9].

Mechanical CPR devices can be alternative tools for maintaining good-quality CPR by bypassing rescuer fatigue, especially in impractical resuscitation situations; therefore, the use of mechanical CPR devices by trained rescuers was recommended in CPR guidelines [3, 4, 10–12]. The simultaneous sterno-thoracic CPR (SST-CPR) device is designed to compress the patient’s sternum directly using a piston and simultaneously straining the patient’s chest wall with a thoracic strap [13]. SST-CPR showed superior hemodynamics, including aortic systolic and diastolic pressures, coronary perfusion pressure (CPP) and end-tidal carbon dioxide (ETCO_2_), and short-term survival, as compared with the standard manual CPR in animal experiments and better ETCO_2_ in a human study [14, 15]. Recently, we developed a new model of an automatic CPR device for performing simultaneous sterno-thoracic CPR that is driven by a battery-powered motor to minimize the inconvenience of using a pneumatic actuator.

We conducted this study to evaluate the hemodynamic effects and resuscitation outcomes of the newly developed, motor-driven, automatic SST-CPR device, in comparison with an automatic mechanical CPR device currently used in clinical practice.

## Materials and methods

### Description of the motor-driven automatic SST-CPR device

**Fig 1:**
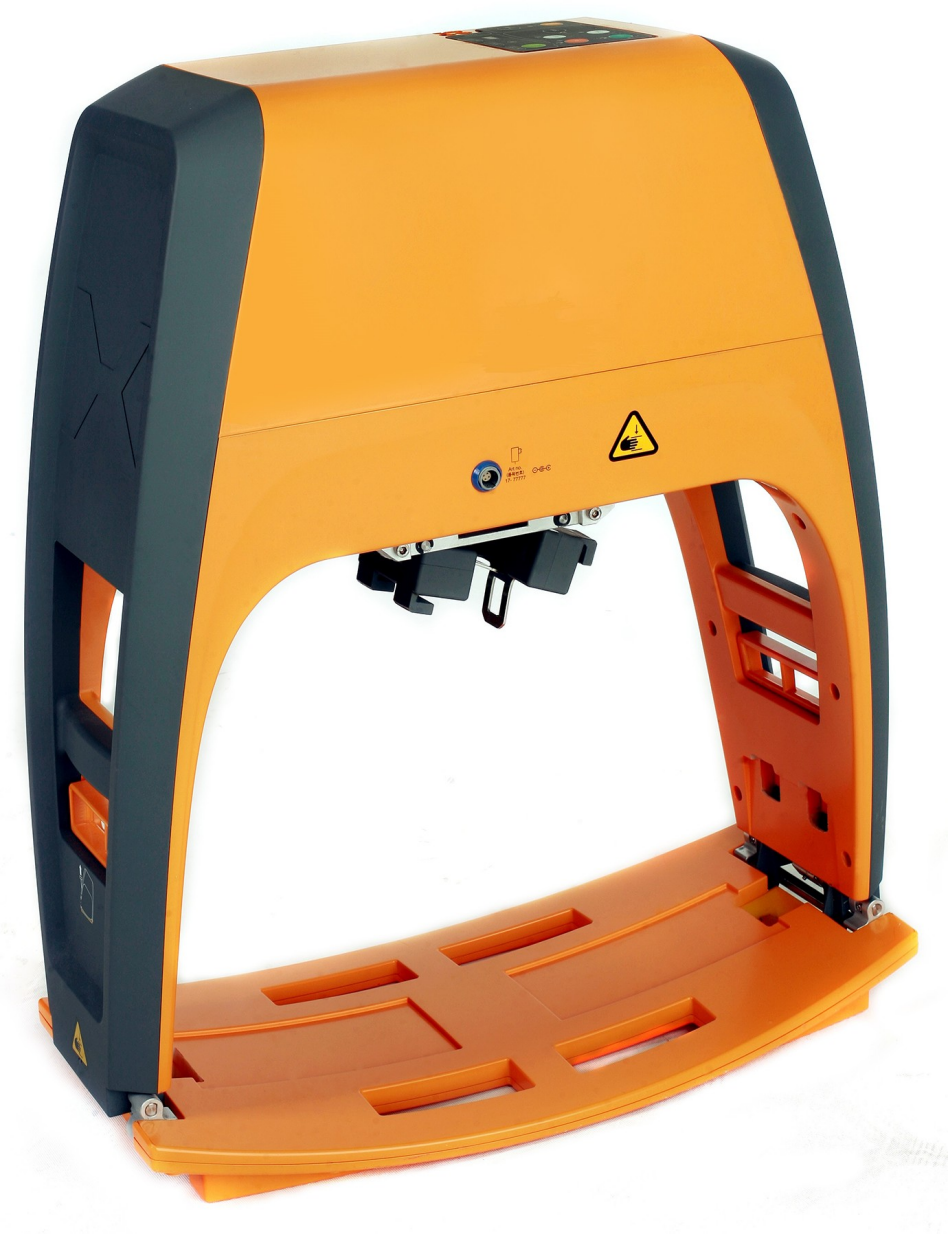
Automatic SST-CPR device (X-CPR). The main compression unit is positioned at the top of the device, which is operated by a battery-powered motor. The strap is attached to both sides of the central piston.

The motor-driven, automatic SST-CPR device (X-CPR 2, CU medical systems, Inc., Wonju, Korea) consists of a piston to compress the patient’s sternum and a thoracic strap to strain the patient’s chest wall while the piston compresses the sternum (S1 and S2 Figs). Additional strain of the thorax is presumed to contribute to a further increase in the pressure of the intrathoracic compartment by preventing configurative changes of the thorax during chest compression. The first version of the automatic SST-CPR device was operated by a pneumatic actuator. Hence, rescuers were required to carry an oxygen tank, thereby precluding the use of the device in places other than hospitals or in places without pressurized oxygen supply. To overcome these disadvantages, we remodeled the device with a motor-driven piston that is powered by an electrical battery (Fig 1).

### Study design and ethical considerations

To evaluate the motor-driven, automatic SST-CPR device, we compared it with the Lund University Cardiac Arrest System, second edition (LUCAS Chest Compression System, Physio-Control, WA), which is commonly used in clinical practice. This study was approved by the Institutional Animal Care and Use Committee of Yonsei University Wonju College of Medicine, Wonju, Republic of Korea (YWC-160324).

### Animal preparation

All study animals were adopted from an institution (Daehan Biolink, Eumseong, Repulic of Korea) accredited from Association for Assessment and Accreditation of Laboratory Animal Care International. Twenty-five Yorkshire pigs (weight 35–60 kg) were used in this study. The pigs were allowed full access to water and food until the day before the experiment and were fasted from midnight. The pigs were initially sedated with intramuscular ketamine (15 mg/kg) and xylazine (2 mg/kg), followed by inhaled 3% isoflurane. After sedation, the pigs were placed in a prone position, and endotracheal intubation was performed with a cuffed endotracheal tube. The animals were then placed in a supine position and ventilated with room air via a volume-controlled ventilator (MDS Matrix 3000, Matrix, Orchard Park, NY). The tidal volume was set at 10 mL/kg, with a ventilation rate of 18 breaths per minute. Intramuscular ketoprofen 1 mg/kg was injected for pain management during the experiment. Electrocardiography (ECG) in lead II and ETCO_2_ were monitored continuously. Under aseptic conditions, the right or left femoral artery was cannulated with a 5.5-Fr introducer sheath using the Seldinger method, and the aortic blood pressures were recorded continuously with a 5-Fr micromanometer-tipped catheter introduced into the femoral artery. An introducer sheath was placed in the right external jugular vein, and the atrial pressure was recorded via a 5-Fr micromanometer-tipped catheter. The right internal carotid artery was exposed, and a vascular flowmeter (Transonic, NY) was applied to monitor the carotid blood flow (CBF). An introducer sheath placed via the right internal jugular vein was used as insertion route for a 5-Fr pacing catheter for inducing VF and infusion of saline and epinephrine. Once the catheters were in place, a 100-unit/kg intravenous (IV) heparin bolus was administered to prevent thrombosis.

### Study protocol

The pigs were randomized into two groups according to the CPR device indicated in a sealed opaque envelope opened by an investigator (KCC) before the induction of cardiac arrest. The envelopes, which contained the name of the CPR device (X-CPR or LUCAS), were randomized by shaking the box and selecting a random envelope from the top of the pile. We observed the pigs without any intervention for 10 minutes after preparation. Baseline hemodynamic data were collected after the observational period. After baseline measurement, a pacing catheter was positioned in the right ventricle. VF was induced by delivering an alternating electrical current at 60 Hz to the endocardium, which was confirmed by the ECG waveform and a decline in aortic pressure. Once VF was induced, the endotracheal tube was disconnected from the ventilator, and the pigs were observed for 2 minutes without any procedure or treatment. After 2 minutes of untreated VF, mimicking the early phase of basic life support (BLS) in which a bystander recognizes cardiac arrest and calls for help, BLS CPR was performed for 6 minutes and advanced cardiac life support (ACLS) CPR was performed for 12 minutes. The chest compression, artificial ventilation, and IV administration of epinephrine were performed in accordance with the guidelines [2–4]. During CPR, the animals received chest compressions using the motor-driven, automatic SST-CPR device or chest compressions with the LUCAS. A total of 30 chest compressions and two consecutive ventilations were performed with the mechanical CPR devices during BLS CPR. The chest compression depth was set at 5 cm, at a rate of 100 per minute. Positive pressure ventilation at approximately 300-mL tidal volume was delivered with a resuscitator bag (Silicone Resuscitator 870040, Laerdal Medical, Stavanger, Norway).

Defibrillation (2 J/kg) was performed after 6 minutes of BLS CPR if the ECG rhythm was shockable, and consecutive defibrillation (4 J/kg) was performed as indicated. During the next 12 minutes of ACLS CPR, chest compression was changed to a continuous mode, and ventilation with 15-L/min oxygen was delivered every 10 chest compressions. One milligram epinephrine in 20-mL saline was administered every 4 minutes until ROSC or until the end of the experiment.

If an animal did not achieve ROSC at 20 minutes after VF induction, the experiment was terminated and the animal was considered dead. When a pig achieved ROSC, we observed it for 2 hours under mechanical ventilation with inhalation anesthesia. After 2 hours, the animal was transferred to the breeding room and observed for 24 hours without post-cardiac arrest care, including targeted temperature management, and intramuscular ketoprofen 1 mg/kg was injected for pain control. Respiratory rate, spontaneous movement, and feeding status were evaluated every 2 hours, and the swine cerebral performance category (CPC) was determined after 24 hours from ROSC, as previously described.[16] In summary, a score of 1 is normal, 2 is mild neurological deficit (e.g., eating or drinking abnormally, unsteady gait, or slight resistance to restraint), 3 is severe neurological deficit (recumbent, unable to stand, and only partially responsive to stimuli), 4 is comatose, and 5 is dead. After the neurological examination, the animals were sedated with intramuscular ketamine and isoflurane inhalation. If the animals had respiratory rate < 5 breaths/min and no response to tactile stimulation, we euthanized with an IV injection of 60-mEq potassium chloride.

We performed autopsy to identify CPR-induced complications at the end of each experiment. The number of rib fracture(s), presence of sternal fracture, pneumothorax, hemothorax, lung contusion, cardiac injury, or great vessel injury was evaluated.

### Measurements

The data were digitized using a digital recording system (PowerLab, ADInstruments, Colorado Springs, CO). Aortic and right atrial pressures, CBF and ETCO_2_ were continuously recorded and analyzed at baseline and every 2 minutes until 20 minutes had elapsed. The CPP during CPR was calculated as the difference between the aortic and right atrial pressures in the mid-diastolic phase using an electrical subtraction unit. Once the pigs achieved ROSC, the measurements of the hemodynamic parameters were stopped because of the possibility of bias from spontaneous circulation.

ROSC was defined as the maintenance of aortic perfusion pressure over 20 minutes. The 24-hour survival rate and swine CPC at 24 hours were evaluated for outcome variables. A favorable neurological outcome was defined as a CPC 1 or 2. The autopsy results were also recorded to compare complications associated with the use of the CPR devices.

### Sample size

On the basis of a previous study that reported a 44-mmHg difference in mean systolic pressure with a standard deviation (SD) of 24 mmHg between the experimental and control groups, at least 12 subjects would be required for both groups to provide a statistical power of 90% with a two-sided alpha value of 0.05 [14]. Twenty-six pigs were chosen, considering that 10% of the animals would be excluded in the analysis owing to unpredictable experimental failure.

### Data analysis

Hemodynamic effects and resuscitation outcomes were compared between the X-CPR and LUCAS groups. Continuous variables were presented as mean ± SD. A Student *t* test or Mann-Whitney *U* test was used to compare the continuous variables between the X-CPR and LUCAS-CPR groups as appropriate. The nominal variables were reported as counts and percentages, and compared using a chi-square or Fisher exact test, as appropriate. A linear mixed model analysis was used to compare hemodynamic parameters, including systolic arterial pressure (SAP), diastolic arterial pressure (DAP), right atrial diastolic pressure (RADP), CBF, CPP, and ETCO_2_ between the two groups. The statistical results are presented as group-time interaction. A p value of <0.05 was considered significant. Analyses were performed using SPSS Statistics 20.0 for Windows (IBM Corp., Chicago, IL, USA).

## Results

### General characteristics and baseline measurements

Thirteen pigs were randomly assigned to each group, and 25 pigs were used in the final analysis because one pig from the LUCAS group died during preparation. The pig had pneumonia and bullous changes on both lungs on autopsy. No significant differences in baseline characteristics and hemodynamic parameters were found between the two groups (Table 1).

**Table 1:** Baseline characteristics and measurements.

Parameters	X-CPR group (n = 13)	LUCAS group (n = 12)	P-value
Body weight (kg)	42±5	46±7	0.087
SAP (mmHg)	90±15	93±31	0.784
DAP (mmHg)	65±15	65±26	0.943
MAP (mmHg)	78±16	74±27	0.642
RAP (mmHg)	5.9±3.7	4.2±2.2	0.156
CBF (ml/min)	358±231	475±305	0.289
CPP (mmHg)	88±14	90±30	0.853
ETCO_2_ (mmHg)	37±6	33±7	0.406

Variables are presented as mean ± standard deviation. X-CPR: sterno-thoracic cardiopulmonary resuscitation device, LUCAS: Lund University Cardiac Arrest System, SAP: systolic arterial pressure, DAP: diastolic arterial pressure, MAP: mean arterial pressure, RAP: right atrial pressure, CBF: carotid blood flow; CPP: coronary perfusion pressure, ETCO_2_: end-tidal carbon dioxide

### Comparison of hemodynamic effects between the X-CPR and LUCAS groups

**Fig 2:**
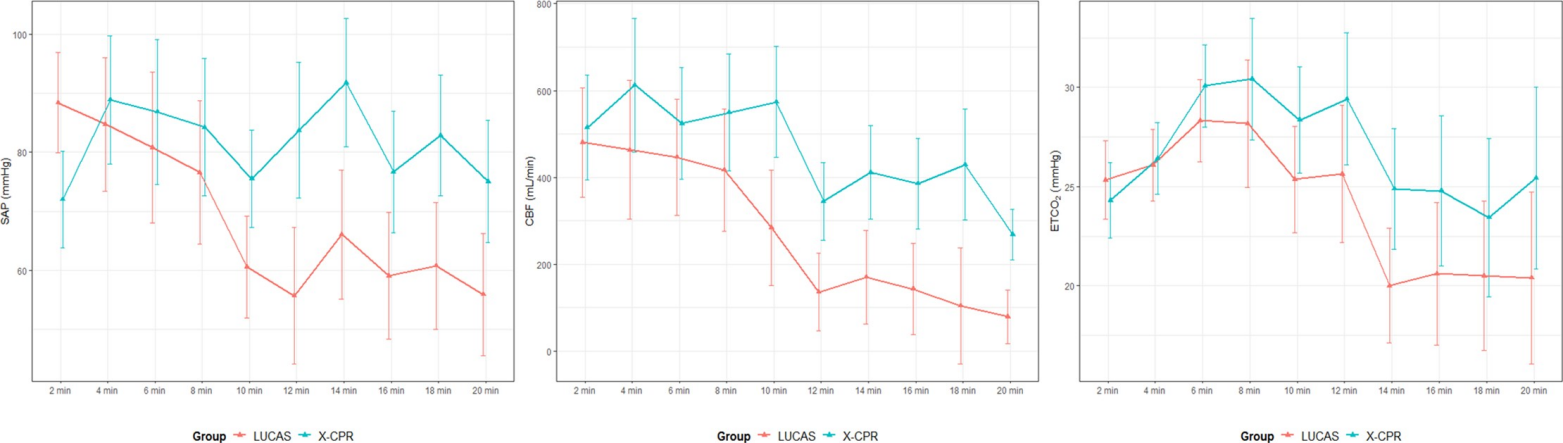
Comparison of hemodynamic effects between the X-CPR and LUCAS groups. SAP: systolic arterial pressure, CBF: carotid blood flow, CPP: coronary perfusion pressure.

We found no significant differences in hemodynamic parameters, including SAP, DAP, RADP, CPP, CBF, and ETCO_2_, between the groups (Table 2). SAP, CBF, and ETCO_2_ tended to be higher in the X-CPR group than in the LUCAS group. However, no statistical significance was observed (Fig 2).

**Table 2:** Comparison of hemodynamic parameters during cardiopulmonary resuscitation.

Parameters	2 min	4 min	6 min	8 min	10 min	12 min	14 min	16 min	18 min	20 min	p-value
**SAP (mmHg)**											0.466
X-CPR	72±20	89±32	87±40	84±42	75±34	84±49	92±38	77±33	83±34	75±34	
LUCAS	88±37	85±46	81±48	77±41	61±24	56±24	66±35	59±35	61±34	56±31	
**DAP (mmHg)**											0.951
X-CPR	21±10	15±11	13±15	12±14	10±13	14±17	23±23	17±20	16±18	12±13	
LUCAS	24±39	22±35	21±30	19±25	17±20	15±12	21±13	12±10	14±9	14±13	
**RADP (mmHg)**											0.918
X-CPR	6.9±4.3	9.2±3.8	8.3±3.8	7.7±3.9	7.4±3.3	7.8±3.3	9.3±4.7	9.3±5.5	11.3±10.6	10.2±9.2	
LUCAS	0.8±8.5	-0.8±8.3	-1.4±7.5	-0.8±7.0	-0.9±7.0	-1.2±7.8	-0.5±7.7	-1.8±7.5	-1.8±7.6	-1.8±7.6	
**CBF (mL/min)**											0.980
X-CPR	515±519	613±698	524±530	550±578	574±582	345±404	412±487	386±481	429±579	269±241	
LUCAS	480±327	463±333	447±376	417±365	274±271	136±118	170±139	143±100	105±81	79±79	
**CPP (mmHg)**											0.994
X-CPR	26±15	26±15	31±31	31±52	43±69	26±25	36±57	36±51	26±43	33±61	
LUCAS	37±30	36±28	27±24	29±19	35±30	31±18	35±35	35±33	24±16	37±46	
**ETCO**_**2**_ **(mmHg)**											0.970
X-CPR	24±5	26±6	30±7	30±8	28±10	29±12	25±8	25±9	23±10	25±12	
LUCAS	24±8	26±7	28±7	28±13	25±8	26±11	20±10	21±3	21±14	20±15	

Data are expressed as mean ± standard deviation. X-CPR, simultaneous sterno-thoracic cardiopulmonary resuscitation; LUCAS, Lund University Cardiac Arrest System; SAP, systolic arterial pressure; DAP, diastolic arterial pressure; RADP, right atrial diastolic pressure; CBF, carotid blood flow; CPP, coronary perfusion pressure; ETCO_2_, end-tidal carbon dioxide

### Comparisons of resuscitation outcomes and complications between the X-CPR and LUCAS groups

Resuscitation outcomes were also not significantly different between the groups. The rate of ROSC was 31% in the X-CPR group and 25% in the LUCAS group (p = 1.000). The 24-hour survival rate was 31% in the X-CPR group and 17% in the LUCAS group (p = 0.645). The 24-hour survival rate with good neurological outcome with a CPC ≤2 was 31% in the X-CPR group and 17% in LUCAS group (p = 0.645; Table 3). A higher incidence of rib fracture with no statistical significance was observed in the X-CPR group as compared with the LUCAS group (69% vs. 33%, p = 0.115). Overall complications detected in postmortem autopsy were also not significantly different between the groups (Table 4).

**Table 3:** Comparison of resuscitation outcomes between the groups.

Parameters	X-CPR (n = 13)	LUCAS (n = 12)	p -value
ROSC, n (%)	4 (31)	3 (25)	1.000
24-hr survival, n (%)	4 (31)	2 (17)	0.645
CPC 1 or 2, n (%)	4 (31)	2 (17)	0.645

Variables are presented as frequency and proportion. X-CPR: Sterno-thoracic cardiopulmonary resuscitation device, LUCAS: Lund University Cardiac Arrest System, ROSC: return of spontaneous circulation, CPC: swine cerebral performance category

**Table 4:** Comparison of complications between the groups.

Parameters	X-CPR (n = 13)	LUCAS (n = 12)	p-value
Rib fractures, n (%)	9 (69)	4 (33)	0.115
Lung contusion	11 (85)	12 (100)	0.480
Hemothorax	0 (0)	2 (17)	0.220
Hemopericardium	1 (8)	0 (0)	1.000
Hemoperitoneum	1 (8)	0 (0)	1.000

Variables are presented as frequency and proportion. X-CPR: Sterno-thoracic cardiopulmonary resuscitation device, LUCAS: Lund University Cardiac Arrest System

## Discussion

High-quality chest compression and minimal interruption are key components for maintaining high-quality CPR during cardiac arrest. Good-quality CPR is difficult to maintain because the quality of chest compression declines during CPR.[17] Mechanical automatic CPR devices can perform CPR without weariness or quality change over time; therefore, they are a good alternative to replace manual chest compressions. They provide high-quality CPR and are better than the standard manual CPR in terms of hemodynamic effects.[18, 19]

We evaluated the hemodynamic effects and resuscitation outcomes of the new X-CPR in comparison with those of the LUCAS, whose hemodynamic and clinical effects have been proven. Mechanical CPR devices use a different mechanism to produce an augmented hemodynamic effect. Therefore, the two different mechanical CPR devices were predicted to have different hemodynamic consequences because X-CPR causes thoracic straining and the LUCAS produces active chest decompressions in addition to chest compressions. The LUCAS is a device commonly used in clinical practice along with AutoPulse (Zoll, Chelmsford, Massachusetts, USA).[20] It compresses and actively decompresses the chest with a suction cup, which improves cardiac output, aortic pressure, cerebral blood flow, and ETCO_2_ by augmenting negative intrathoracic pressure and increasing venous return.[21] The hemodynamic superiority of the LUCAS over the manual CPR has been shown in clinical studies and would be an important reason for the worldwide use of the LUCAS.[22, 23] X-CPR is designed to augment systolic blood flow by straining the chest wall, in addition to the piston-derived chest compression.[13] It generates higher mean aortic pressure, CPP, and ETCO_2_, and improves short-term survival as compared with the standard manual CPR in animal studies; augmented CPP with X-CPR was also reported in a clinical study.[13–15] The first version of X-CPR was operated with a pneumatic actuator, hence requiring pressurized oxygen or air. Owing to the need for pressurized oxygen, emergency medical personnel should bring an oxygen tank when they use X-CPR in prehospital settings. The second version of X-CPR, which is a motor-driven, battery-powered automatic CPR device, does not require an oxygen tank. The study results showed that CPR using X-CPR tended to maintain higher SBP, ETCO_2_, and CBF, which is an indicator of systemic and cerebral perfusions, than CPR with the LUCAS, although the difference did not reach statistical significance. These findings would be the result of the synchronous compression effect of the central piston and circumferential strap, and the augmented thoracic filling from the negative right atrial pressure during the early relaxation phase, which were reported in a previous study.[13] The results of this study demonstrated that X-CPR and the LUCAS have at least a similar hemodynamic effects and resuscitation outcomes.

Better resuscitation outcomes, including 24-hour survival and favorable neurological outcome, were observed in the X-CPR group, although the differences were not statistically significant. The hemodynamic-directed CPR targeting SBPs > 90 mmHg was introduced recently and showed better survival and neurological outcome than the conventional CPR method targeting the designated compression depth and rate.[24, 25] These findings imply that the hemodynamics during CPR would be more important than keeping a constant compression depth and rate for improving resuscitation outcomes. Our study showed that higher SAP was maintained in the X-CPR group than in the LUCAS group, and would be a major reason for the better outcomes in the former group.

In our study, both X-CPR and the LUCAS were associated with high incidence rates of lung contusions and rib fractures. This result raised a concern on the high rate of complications with the mechanical CPR devices. The frequency of rib fracture was higher in the X-CPR group than in the LUCAS group, although the difference was statistically insignificant. In a study to compare safety between the LUCAS, AutoPulse, and manual CPR, the incidence rates of rib and sternal damages were not statistically different between the CPR methods, although the rates tended to be higher in AutoPulse than in the LUCAS.[26] This would be a consequence of the use of a circumferential band such as X-CPR to strain the thorax in AutoPulse. The high incidence of rib fractures in X-CPR may be associated with the use of a thoracic strap as in AutoPulse. The high incidence of rib fractures in both X-CPR and the LUCAS may be partly due to the difference in the anatomic conformation of the chest between animals and humans.

Mechanical CPR has not shown superiority over manual CPR in terms of survival or favorable neurological outcomes in large clinical trials.[27, 28] However, generalization of the results from clinical trials is limited because the extremely high manual CPR performance of EMS systems included in clinical trials might not reflect the average performance of an EMS system. Mechanical CPR offers undoubtedly a high quality of resuscitation even in special situations, including ambulance or helicopter transport.[29, 30] Mechanical CPR devices can be used for maintaining systemic perfusion during percutaneous coronary intervention, pericardiocentesis, or extracorporeal membrane oxygenation in patients with circulatory collapse or cardiac arrest.[31, 32] In this respect, further study of clinical situations where mechanical CPR is effective is needed.

This study has several limitations. First, we used an animal cardiac arrest model; therefore, the study results would be difficult to extrapolate to human individuals. The thoracic conformation in pigs is more oval than that in humans; thus, the hemodynamic effects produced by the thoracic strap of X-CPR or the suction cup of the LUCAS may be different from those in human beings. The accompanying limitations of an experimental animal model include the mode and cause of cardiac arrest that differ from those in clinical situations, the use of a controlled environment in the experimental setting, and the use of anesthetics. The low numbers of resuscitated pigs and high incidence of complications that might reflect a conformational difference of the chest wall from that of humans warrants a need for future studies on X-CPR to generalize this result. Second, even though the investigators tried to maintain approximately 300 mL of ventilation volume by squeezing one-third of a resuscitation bag with a volume of 1,500 mL, the delivered volume in every ventilation might be different and could influence the hemodynamic parameters during CPR by affecting the intrathoracic pressure and volume. Third, histopathological evaluation to determine the brain outcome was not included in this study because it was designed to compare hemodynamic effects and outcomes between two CPR devices. Finally, the sample size was too small to determine the difference between the groups, although it was calculated on the basis of a previous study.

## Conclusions

CPR with a motor-driven X-CPR and CPR with the LUCAS produced similar hemodynamic effects and resuscitation outcomes in a swine model of cardiac arrest.

## Supporting information

S1 Fig Mechanism of simultaneous sternothoracic cardiopulmonary resuscitation with X-CPR illustrated on an axial image of the chest. X-CPR exploits compression of the sternum with a piston (black arrow) and simultaneous constriction of the thorax with a strap (gray arrow) in a cycle.
(GIF)

S2 Fig Animated illustration of simultaneous sternothoracic cardiopulmonary resuscitation. (GIF)

## References

[1] Benjamin EJ, Virani SS, Callaway CW, Chamberlain AM, Chang AR, Cheng S, et al Heart Disease and Stroke Statistics-2018 Update: A Report From the American Heart Association. Circulation. 2018;137:e67–e492. 10.1161/CIR.0000000000000558 29386200

[2] Kleinman ME, Brennan EE, Goldberger ZD, Swor RA, Terry M, Bobrow BJ, et al Part 5: Adult Basic Life Support and Cardiopulmonary Resuscitation Quality: 2015 American Heart Association Guidelines Update for Cardiopulmonary Resuscitation and Emergency Cardiovascular Care. Circulation. 2015;132:S414–35. 10.1161/CIR.0000000000000259 26472993

[3] Soar J, Nolan JP, Bottiger BW, Perkins GD, Lott C, Carli P, et al European Resuscitation Council Guidelines for Resuscitation 2015: Section 3. Adult advanced life support. Resuscitation. 2015;95:100–47. 10.1016/j.resuscitation.2015.07.016 26477701

[4] Lee MJ, Rho TH, Kim H, Kang GH, Kim JS, Rho SG, et al Part 3. Advanced cardiac life support: 2015 Korean Guidelines for Cardiopulmonary Resuscitation. Clinical and experimental emergency medicine. 2016;3:S17–s26. 10.15441/ceem.16.134 27752643 PMC5052917

[5] Kashiura M, Hamabe Y, Akashi A, Sakurai A, Tahara Y, Yonemoto N, et al Association between cardiopulmonary resuscitation duration and one-month neurological outcomes for out-of-hospital cardiac arrest: a prospective cohort study. BMC anesthesiology. 2017;17:59 10.1186/s12871-017-0351-1 28431508 PMC5401557

[6] Perkins GD, Augre C, Rogers H, Allan M, Thickett DR. CPREzy: an evaluation during simulated cardiac arrest on a hospital bed. Resuscitation. 2005;64:103–8. 10.1016/j.resuscitation.2004.08.011 15629562

[7] Wik L, Kramer-Johansen J, Myklebust H, Sorebo H, Svensson L, Fellows B, et al Quality of cardiopulmonary resuscitation during out-of-hospital cardiac arrest. Jama. 2005;293:299–304. 10.1001/jama.293.3.299 15657322

[8] Idris AH, Guffey D, Aufderheide TP, Brown S, Morrison LJ, Nichols P, et al Relationship between chest compression rates and outcomes from cardiac arrest. Circulation. 2012;125:3004–12. 10.1161/CIRCULATIONAHA.111.059535 22623717 PMC3388797

[9] Christenson J, Andrusiek D, Everson-Stewart S, Kudenchuk P, Hostler D, Powell J, et al Chest compression fraction determines survival in patients with out-of-hospital ventricular fibrillation. Circulation. 2009;120:1241–7. 10.1161/CIRCULATIONAHA.109.852202 19752324 PMC2795631

[10] Wang PL, Brooks SC. Mechanical versus manual chest compressions for cardiac arrest. The Cochrane database of systematic reviews. 2018;8:Cd007260 10.1002/14651858.CD007260.pub4 30125048 PMC6953326

[11] Bonnes JL, Brouwer MA, Navarese EP, Verhaert DV, Verheugt FW, Smeets JL, et al Manual Cardiopulmonary Resuscitation Versus CPR Including a Mechanical Chest Compression Device in Out-of-Hospital Cardiac Arrest: A Comprehensive Meta-analysis From Randomized and Observational Studies. Annals of emergency medicine. 2016;67:349–60.e3. 10.1016/j.annemergmed.2015.09.023 26607332

[12] Brooks SC, Anderson ML, Bruder E, Daya MR, Gaffney A, Otto CW, et al Part 6: Alternative Techniques and Ancillary Devices for Cardiopulmonary Resuscitation: 2015 American Heart Association Guidelines Update for Cardiopulmonary Resuscitation and Emergency Cardiovascular Care. Circulation. 2015;132:S436–43. 10.1161/CIR.0000000000000260 26472994

[13] Hwang SO, Lee KH, Cho JH, Oh BJ, Gupta DS, Ornato JP, et al Simultaneous sternothoracic cardiopulmonary resuscitation: a new method of cardiopulmonary resuscitation. Resuscitation. 2001;48:293–9. 11278095 10.1016/s0300-9572(00)00250-1

[14] Hwang SO, Lee KH, Lee JW, Lee SY, Yoo BS, Yoon J, et al Simultaneous sterno-thoracic cardiopulmonary resuscitation improves short-term survival rate in canine cardiac arrests. Resuscitation. 2002;53:209–16. 12009225 10.1016/s0300-9572(02)00011-4

[15] Lee DK, Cha YS, Kim OH, Cha KC, Lee KH, Hwang SO, et al Effect of Automated Simultaneous Sternothoracic Cardiopulmonary Resuscitation Device on Hemodynamics in Out-of-Hospital Cardiac Arrest Patients. The Journal of emergency medicine. 2018;55:226–34. 10.1016/j.jemermed.2018.04.060 29885734

[16] Berg RA, Otto CW, Kern KB, Sanders AB, Hilwig RW, Hansen KK, et al High-dose epinephrine results in greater early mortality after resuscitation from prolonged cardiac arrest in pigs: a prospective, randomized study. Critical care medicine. 1994;22:282–90. 10.1097/00003246-199402000-00020 8306688

[17] Hightower D, Thomas SH, Stone CK, Dunn K, March JA. Decay in quality of closed-chest compressions over time. Annals of emergency medicine. 1995;26:300–3. 10.1016/s0196-0644(95)70076-5 7661418

[18] Duchateau FX, Gueye P, Curac S, Tubach F, Broche C, Plaisance P, et al Effect of the AutoPulse automated band chest compression device on hemodynamics in out-of-hospital cardiac arrest resuscitation. Intensive care medicine. 2010;36:1256–60. 10.1007/s00134-010-1784-x 20213073 PMC2929359

[19] Rubertsson S, Karlsten R. Increased cortical cerebral blood flow with LUCAS; a new device for mechanical chest compressions compared to standard external compressions during experimental cardiopulmonary resuscitation. Resuscitation. 2005;65:357–63. 10.1016/j.resuscitation.2004.12.006 15919574

[20] Nolan JP. High-quality cardiopulmonary resuscitation. Current opinion in critical care. 2014;20:227–33. 10.1097/MCC.0000000000000083 24717696

[21] Steen S, Liao Q, Pierre L, Paskevicius A, Sjoberg T. Evaluation of LUCAS, a new device for automatic mechanical compression and active decompression resuscitation. Resuscitation. 2002;55:285–99. 12458066 10.1016/s0300-9572(02)00271-x

[22] Axelsson C, Karlsson T, Axelsson AB, Herlitz J. Mechanical active compression-decompression cardiopulmonary resuscitation (ACD-CPR) versus manual CPR according to pressure of end tidal carbon dioxide (P(ET)CO2) during CPR in out-of-hospital cardiac arrest (OHCA). Resuscitation. 2009;80:1099–103. 10.1016/j.resuscitation.2009.08.006 19716640

[23] Larsen AI, Hjornevik A, Bonarjee V, Barvik S, Melberg T, Nilsen DW. Coronary blood flow and perfusion pressure during coronary angiography in patients with ongoing mechanical chest compression: a report on 6 cases. Resuscitation. 2010;81:493–7. 10.1016/j.resuscitation.2010.02.002 20227005

[24] Friess SH, Sutton RM, Bhalala U, Maltese MR, Naim MY, Bratinov G, et al Hemodynamic directed cardiopulmonary resuscitation improves short-term survival from ventricular fibrillation cardiac arrest. Critical care medicine. 2013;41:2698–704. 10.1097/CCM.0b013e318298ad6b 23887237 PMC3812371

[25] Lautz AJ, Morgan RW, Karlsson M, Mavroudis CD, Ko TS, Licht DJ, et al Hemodynamic-Directed Cardiopulmonary Resuscitation Improves Neurologic Outcomes and Mitochondrial Function in the Heart and Brain. Critical care medicine. 2019;47:e241–e9. 10.1097/CCM.0000000000003620 30779720 PMC6561502

[26] Koster RW, Beenen LF, van der Boom EB, Spijkerboer AM, Tepaske R, van der Wal AC, et al Safety of mechanical chest compression devices AutoPulse and LUCAS in cardiac arrest: a randomized clinical trial for non-inferiority. European heart journal. 2017;38:3006–13. 10.1093/eurheartj/ehx318 29088439 PMC5837501

[27] Rubertsson S, Lindgren E, Smekal D, Ostlund O, Silfverstolpe J, Lichtveld RA, et al Mechanical chest compressions and simultaneous defibrillation vs conventional cardiopulmonary resuscitation in out-of-hospital cardiac arrest: the LINC randomized trial. Jama. 2014;311:53–61. 10.1001/jama.2013.282538 24240611

[28] Perkins GD, Lall R, Quinn T, Deakin CD, Cooke MW, Horton J, et al Mechanical versus manual chest compression for out-of-hospital cardiac arrest (PARAMEDIC): a pragmatic, cluster randomised controlled trial. Lancet (London, England). 2015;385:947–55. 10.1016/S0140-6736(14)61886-9 25467566

[29] Russi CS, Myers LA, Kolb LJ, Lohse CM, Hess EP, White RD. A Comparison of Chest Compression Quality Delivered During On-Scene and Ground Transport Cardiopulmonary Resuscitation. The western journal of emergency medicine. 2016;17:634–9. 10.5811/westjem.2016.6.29949 27625733 PMC5017853

[30] Rehatschek G, Muench M, Schenk I, Dittrich W, Schewe JC, Dirk C, et al Mechanical LUCAS resuscitation is effective, reduces physical workload and improves mental performance of helicopter teams. Minerva anestesiologica. 2016;82:429–37. 26576860

[31] Wagner H, Hardig BM, Rundgren M, Zughaft D, Harnek J, Gotberg M, et al Mechanical chest compressions in the coronary catheterization laboratory to facilitate coronary intervention and survival in patients requiring prolonged resuscitation efforts. Scandinavian journal of trauma, resuscitation and emergency medicine. 2016;24:4 10.1186/s13049-016-0198-3 26795941 PMC4721004

[32] William P, Rao P, Kanakadandi UB, Asencio A, Kern KB. Mechanical Cardiopulmonary Resuscitation In and On the Way to the Cardiac Catheterization Laboratory. Circulation journal: official journal of the Japanese Circulation Society. 2016;80:1292–9. 27180892 10.1253/circj.CJ-16-0330

