# Supplementary figures and images for: Comparison of hemodynamic effects and resuscitation outcomes between automatic simultaneous sterno-thoracic cardiopulmonary resuscitation device and LUCAS in a swine model of cardiac arrest
Source: PLoS One. 2019 Aug 30;14(8):e0221965. doi: 10.1371/journal.pone.0221965 (PMC6716643; doi:10.1371/journal.pone.0221965)

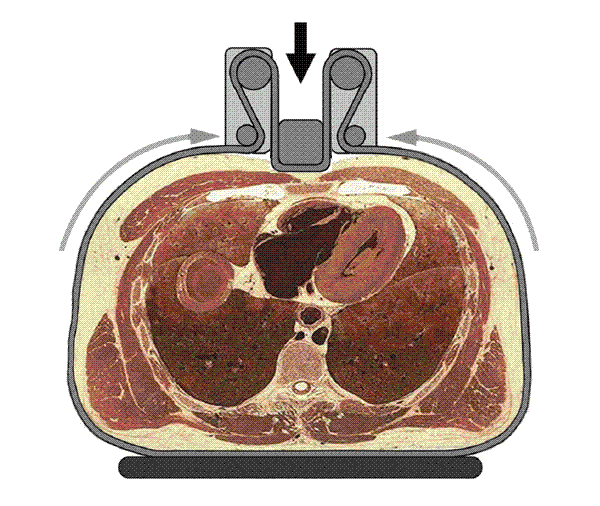

Supplement: S1 Fig — X-CPR exploits compression of the sternum with a piston (black arrow) and simultaneous constriction of the thorax with a strap (gray arrow) in a cycle. (GIF) [file pone.0221965.s001.GIF]

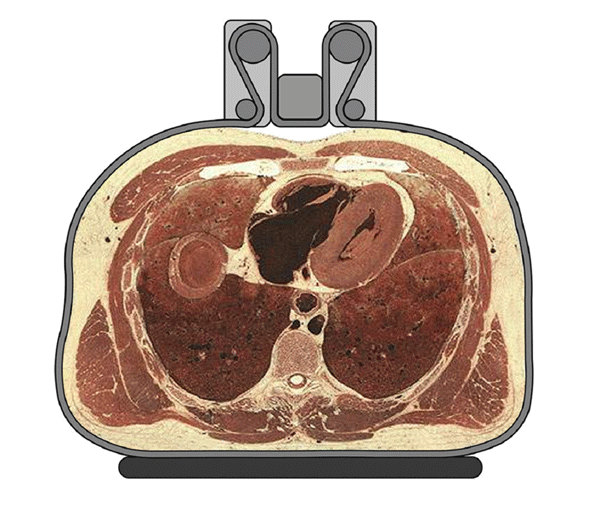

Supplement: S2 Fig — (GIF) [file pone.0221965.s002.gif]
